# Association of urinary incontinence with depression among men: a cross-sectional study

**DOI:** 10.1186/s12889-023-15961-9

**Published:** 2023-05-25

**Authors:** Shasha Wu, Feixiang Wu

**Affiliations:** 1 Department of Gastroenterology, The Third Hospital of Mianyang, Sichuan Mental Health Center, Mianyang, Sichuan People’s Republic of China; 2 Department of Urology, The Third Hospital of Mianyang, Sichuan Mental Health Center, Mianyang, Sichuan People’s Republic of China

**Keywords:** Urinary incontinence, Depression, Cross-sectional study, Association, NHANES

## Abstract

**Objective:**

Depression and urinary incontinence (UI) are both troubling symptoms that severely impact quality of life. The aim of this study is to evaluate the association between UI (including UI types and severity) and depression among men.

**Population and methods:**

The analyzed data was collected from the 2005–2018 National Health and Nutrition Examination Survey (NHANES) data. A total of 16,694 male participants aged ≥ 20 years with complete information about depression and UI were included in this study. Logistic regression was performed to calculate the odds ratio (OR) and 95% confidence interval (CI) to determine the association between depression and UI by adjusting for relevant covariables.

**Results:**

The prevalence of depression was 10.91% among participants with UI. Urge UI was the main type of UI and accounts for 50.53% of all UI types. The adjusted ORs for the association between depression and UI were 2.69 (95%CI, 2.20–3.28). Compared with slight UI, the adjusted ORs were 2.28 (95% CI, 1.61–3.23) for moderate UI, 2.98 (95% CI, 1.54–5.74) for severe UI, and 3.85 (95% CI, 1.83–8.12) for very severe UI. Compared with no UI, the adjusted ORs were 4.46 (95% CI, 3.16–6.29) for mixed UI, 3.15 (95% CI, 2.06–4.82) for stress UI, and 2.43 (95% CI, 1.89–3.12) for urge UI. The subgroup analyses also showed similar correlation about depression and UI.

**Conclusion:**

Among men, depression was positively associated with UI status, severity and types. For clinicians, it’s necessary to screen depression in patients with UI.

## Introduction

Urinary incontinence (UI) is a common symptom in adults that does not threaten survival but significantly affects the quality of life of patients. UI types are defined as three main types: stress UI (SUI, urine loss during movement or efforts or in general during increased abdominal pressure), urge UI (UUI, urine loss concomitant or immediately following an urgency episode), and mixed UI (MUI, any combination of SUI and UUI) [1, 2]. About 38.5% of US men over the age of 60 have UI [3], and the prevalence appears to increase with age [4]. UUI accounts for the majority of UI, about 40–80%, and is also the type that causes the most trouble for male UI patients [1, 5]. The etiology of male UI has been reported including age, neurological causes, overactive bladder syndrome, physical limitation, prostate surgery, obesity, etc. [1, 6–9].

Mental health is an important part of human health. Wellness means not only the absence of disease or disability, but also physical, mental and social health. It also represents the quality of life. Except for objective biological indicators, doctors need to consider the subjective evaluation of the patients’ own health when assessing the health status. It has been observed that mental health is significantly correlated with physical health [10]. Depression is a common and serious mental health problem around the world [11]. The prevalence of major depression for lifetime is up to 16.2% [12]. In addition, depression is a main factor of suicide and one of the main causes of death [13].

The relationship between depression and UI has attracted doctors’ attention. It has been mentioned that depression is associated with lower urinary tract symptoms [14], especially UI, which has been reported as a risk factor for depression [15, 16]. However, few studies have systematically reported the association between depression and different UI types and severity in men. In this cross-sectional study, we reported the independent roles of UI, UI severity, and UI types in depression among adult men.

## Methods

### Study design and eligibility criteria

Data analyzed in this study was collected from the National Health and Nutrition Examination Survey (NHANES, https://www.cdc.gov/nchs/nhanes/) from 2005 to 2018. NHANES is a program combining interviews and physical examinations to assess the health and nutritional status of the U.S. population. Participants were selected through a multistage probability sampling to be representative of the entire U.S. population. The interviews were conducted at the participants’ homes. The examinations were performed at the mobile center. The public and deidentified information of participants was collected.

**Fig. 1:**
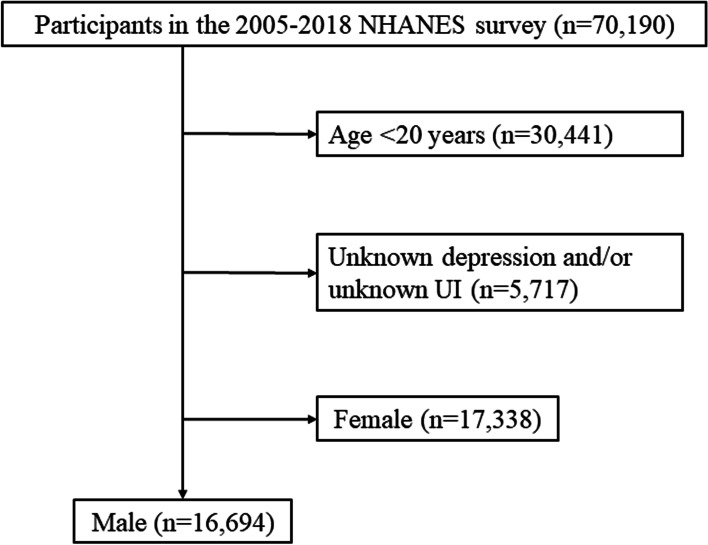
Flowchart showing the selection of participants

There were 70,190 participants in the 2005–2018 NHANES program. Participants who were younger than 20 years (30,441), with incomplete information about depression and/or UI (5,717), or were female (17,338) were excluded. A total of 16,694 male participants were included in this study. A flowchart describing how to select participants is shown in Fig. 1.

### Definition of depression

Depression symptoms were determined by the Patient Health Questionnaire (PHQ-9) screening instrument, which consists of nine symptom questions and showed excellent sensitivity and specificity in assessing depression status [17]. Response to each question is divided into “not at all”, “several days”, “more than half the days” and “nearly every day”, with 0 to 3 points respectively. The total score of each participant was calculated by aggregating scores from nine questions, with a score ≥ 10 defined as depression.

### Assessment of UI

The UI was assessed based on the information collected from the kidney condition questionnaire. When participants answer “never” to the question “How often have urinary leakage?”, it was defined as having no UI, otherwise it was UI.

The severity index for UI was obtained by multiplying frequency (four levels) and amount of leakage (three levels), according to questions “How often have urinary leakage?” and “How much urine lose each time?” separately [18]. Then, the UI severity was divided into four levels based on the severity index values: slight (1–2), moderate (3–6), severe (8–9), and very severe (10–12).

The types of UI were defined according to the following questions: participants answered “yes” to “Leak urine during physical activities?” were defined as suffering from SUI; those who answered “yes” to “Urinated before reaching the toilet?” were defined as suffering from UUI; those who answered “yes” to both questions were defined as suffering from MUI; participants that cannot be included in SUI, UUI or MUI were defined as suffering from other type of UI.

### Covariates

The covariates were collected from demographics, examination and questionnaire data, including age, race/ethnicity, marital status, education, health insurance, family income, smoking status, alcohol consumption, body mass index (BMI) and physical activity. Age was classified as < 40, 40–59 and ≥ 60 years. Race/ethnicity was classified as Mexican American, non-Hispanic White, non-Hispanic Black, other Hispanic and other races. Marital status was categorized as never married, married/living with partner, and widowed/divorced/separated. Education level was divided into < high school, high school and > high school. Health insurance was defined based on self-reported insurance data and was classified as yes and no. Family income was evaluated with ratio of family income to poverty (RIP) and divided into three levels: poverty (0–1.00), moderate (1.01–3.00), rich (> 3.00). Participants who smoked at least 100 cigarettes in life were defined as smoking. Then, these participants were divided into current smoker and former smoker, based on the question: “Do you now smoke cigarettes?”. Participants who had at least 12 alcohol drinks/one year or lifetime were defined as drinking alcohol, otherwise they did not drink alcohol. BMI was used to assess adiposity status and classified as < 25.0, 25.0–29.9 and ≥ 30.0 kg/m^2^. Metabolic equivalent (MET-min/week) was used to evaluate physical activity and divided into insufficient activity (MET < 500) and enough activity (MET ≥ 500) [19, 20]. Missing data in covariates were categorized as unknown.

### Statistical analyses

Taking into account the multistage probability sampling in NHANES, sample weights, clustering and strata were applied to all analyses was performed according to the NHANES Analytic Guidelines (https://wwwn.cdc.gov/nchs/nhanes/analyticguidelines.aspx). Characteristics of participants were stratified by UI (no/yes) and assessed with chi-squared test for all variables. The logistic regression analysis was conducted to estimate the correlation between depression status and UI by calculating the odds ratio (OR) and 95% confidence interval (CI). Three multivariate regression models were constructed: model 1, non-adjusted; model 2, age and race/ethnicity were adjusted; model 3, all covariates were adjusted. In addition, subgroup analysis was performed to assess the UI-depression correlation stratified by age, race/ethnicity, marital status, education, health insurance, smoking, alcohol consumption, PIR, BMI, and MET. All statistical analyses were performed using R and EmpowerStats, and the statistical significance criterion was set at *P* < 0.05.

## Results

### Characteristics of participants in this study

Overall, 16,694 participants were included in this study, consisting of 1,096 participants suffering from depression and 4,038 participants suffering from UI. Characteristics of participants are shown in Table 1. A total of 10.91% of UI participants suffered from depression at the same time. As shown in Table 1, 43.56% of UI participants had slight symptom and 25.41% had moderate to severe symptom. UUI is the main type of UI, accounting for 50.53% of all UI, while SUI accounts for 9.41% and MUI accounts for 10.19%. Participants with UI were more likely than those without UI to be older (48.14% vs 18.38%), to be non-Hispanic White or Black (82.68% vs 76.96%), widowed (18.20% vs 11.71%), less than high school (19.49% vs 15.57%), have moderate household income (34.54% vs 32.23%), have health insurance (85.22% vs 78.46%), former smoker (37.90% vs 27.38%), higher BMI (41.46% vs 34.53%), lower MET (30.41% vs 21.33%).

**Table 1:** Baseline characteristics of male participants ≥ 20 years old in the 2005–2018 NHANES

	**UI = No**		**UI = Yes**		
**Variables**^a^	**N**	**percentage**	**N**	**percentage**	***P*-value**
**Depression**					< 0.0001
No	12,042	95.67	3556	89.09	
Yes	614	4.33	482	10.91	
**UI severity**					
Slight		0.00	1610	43.56	
Moderate		0.00	872	20.52	
Severe		0.00	140	2.77	
Very severe		0.00	112	2.12	
Unknown	12,656	100.00	1304	31.03	
**UI type**					
No	12,656	100.00		0.00	
SUI		0.00	344	9.41	
UUI		0.00	2185	50.53)	
MUI		0.00	452	10.19	
Other		0.00	1057	29.87	
**Age (mean and SD)**	12,656	46.43 (17.18)	4038	60.20 (15.67)	< 0.0001
**Age (years)**^b^					< 0.0001
< 40	5086	43.36	512	15.75	
40–59	4225	38.26	1133	36.11	
≥ 60	3345	18.38	2393	48.14	
**Race/Ethnicity**					< 0.0001
Mexican American	2076	9.67	523	7.09	
Non-Hispanic White	5393	67.23	1865	70.19	
Non-Hispanic Black	2510	9.73	1057	12.49	
Other Hispanic	1160	5.66	307	4.37	
Other races	1517	7.71	286	5.86	
**Marital status**					< 0.0001
Never married	2637	21.71	496	12.51	
Married/living with partner	8238	66.57	2615	69.18	
Widowed/divorced/separated	1777	11.71	922	18.20	
Unknown	4	0.02	5	0.10	
**Education**^b^					< 0.0001
< High school	3072	15.57	1151	19.49	
High school	3053	24.37	980	24.48	
> High school	6526	60.01	1903	55.94	
Unknown	5	0.05	4	0.08	
**RIP**^b^					< 0.0001
0–1.00	2253	12.04	717	11.68	
1.01–3.00	4817	32.23	1639	34.54	
> 3.00	4562	49.34	1314	45.86	
Unknown	1024	6.39	368	7.93	
**Health insurance**					< 0.0001
No	3318	21.47	632	14.75	
Yes	9325	78.46	3404	85.22	
Unknown	13	0.07	2	0.02	
**Smoking**					< 0.0001
No	6115	49.99	1454	39.07	
Former smoker	3467	27.38	1604	37.90	
Current smoker	3066	22.61	979	23.01	
Unknown	8	0.03	1	0.03	
**Alcohol consumption**					< 0.0001
No	909	5.92	213	4.69	
Yes	10,117	79.43	3097	75.80	
Unknown	1630	14.65	728	19.51	
**BMI**^b^					< 0.0001
< 25.0	3522	26.34	941	22.35	
25.0–29.9	4896	38.52	1402	34.70	
≥ 30.0	4142	34.53	1617	41.46	
Unknown	96	0.61	78	1.49	
**MET**^b^					< 0.0001
< 500	3177	21.33	1464	30.41	
≥ 500	7839	64.88	2114	56.82	
Unknown	1640	13.79	460	12.78	

*Abbreviations*: *N* frequency of participants, *UI* urinary incontinence, *SUI* stress UI, *UUI* urge UI, *MUI* mixed UI, *RIP* ratio of family income to poverty, *BMI* body mass index, *MET* metabolic equivalent

^a^Variables are presented as frequency and weighted percentage and compared by Chi-square test

^b^ Variables are presented as frequency and weighted percentage and compared by Wilcoxon rank sum test

### Association between UI and depression

Multivariate logistic regression analysis was performed and three models were constructed to test the association between UI and depression (Table 2). Participants with UI had a 2.70-fold higher risk of depression compared with participants without UI in unadjusted model. In full adjusted model, this OR was attenuated to be 2.69 (95%CI, 2.20–3.28).

**Table 2:** Association of depression with urinary incontinence among male participants ≥ 20 years old in 2005–2018 NHANES

	**Non-adjusted**		**Adjust I**		**Adjust II**	
	**OR (95%CI)**	***P*-value**	**OR (95%CI)**	***P*-value**	**OR (95%CI)**	***P*-value**
**Male**						
**UI**						
No	Ref		Ref		Ref	
Yes	2.70 (2.29, 3.20)	< 0.0001	3.10 (2.57, 3.74)	< 0.0001	2.69 (2.20, 3.28)	< 0.0001
**UI severity**						
Slight	Ref		Ref		Ref	
Moderate	2.05 (1.48, 2.83)	< 0.0001	2.26 (1.64, 3.12)	< 0.0001	2.28 (1.61, 3.23)	< 0.0001
Severe	2.59 (1.43, 4.71)	0.0023	2.90 (1.59, 5.29)	0.0007	2.98 (1.54, 5.74)	0.0017
Very severe	4.01 (2.10, 7.66)	0.0001	4.81 (2.58, 8.98)	< 0.0001	3.85 (1.83, 8.12)	0.0007
Unknown	0.51 (0.42, 0.63)	< 0.0001	0.45 (0.36, 0.57)	< 0.0001	0.50 (0.40, 0.64)	< 0.0001
**UI type**						
No	Ref		Ref		Ref	
SUI	3.10 (2.16, 4.46)	< 0.0001	3.46 (2.38, 5.03)	< 0.0001	3.15 (2.06, 4.82)	< 0.0001
UUI	2.43 (1.95, 3.03)	< 0.0001	2.84 (2.22, 3.62)	< 0.0001	2.43 (1.89, 3.12)	< 0.0001
MUI	5.04 (3.67, 6.91)	< 0.0001	6.37 (4.55, 8.92)	< 0.0001	4.46 (3.16, 6.29)	< 0.0001
Other	2.32 (1.74, 3.11)	< 0.0001	2.62 (1.95, 3.54)	< 0.0001	2.44 (1.77, 3.36)	< 0.0001

Non-adjusted model adjusted for: none

Adjust I model adjusted for: age, race/ethnicity

Adjust II model adjusted for: age, race/ethnicity, marital status, education, health insurance, smoking, alcohol consumption, PIR, BMI, MET

*Abbreviations*: *OR* odds ratio, *CI* confidence interval, *UI* urinary incontinence, *SUI* stress UI, *UUI* urge UI, *MUI* mixed UI, *RIP* ratio of family income to poverty, *BMI* body mass index, *MET* metabolic equivalent

UI severity was positively associated with depression. Compared with slight UI, higher odds of depression can be found in men suffered from moderate UI (2.28-fold), severe UI (2.98-fold), and very severe UI (3.85-fold) in full adjusted model. Among all types of UI, MUI participants faced higher odds of depression. Compared with no UI, higher odds of depression can be found in men suffered from MUI (4.46-fold), SUI (3.15-fold), and UUI (2.43-fold) after full adjustment. Similar ORs were also observed in unadjusted and partial adjusted models.

**Fig. 2:**
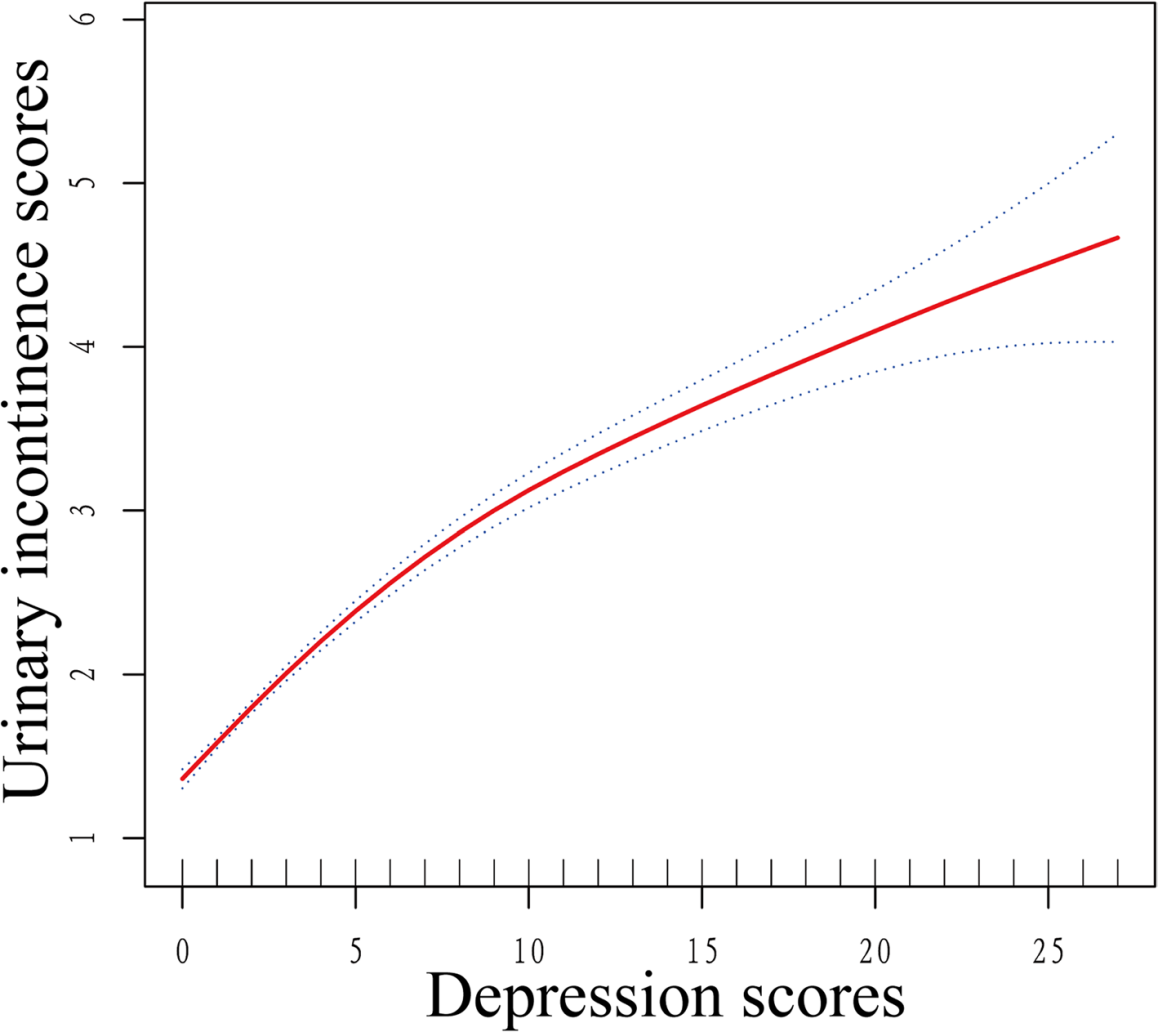
Smooth curve of the trend of the relationship between UI and depression

Addition, a smooth curve was conducted to reveal the trend of the association between UI and depression. The results showed that higher depression scores related to higher UI scores (Fig. 2).

### Subgroup analysis

**Fig. 3:**
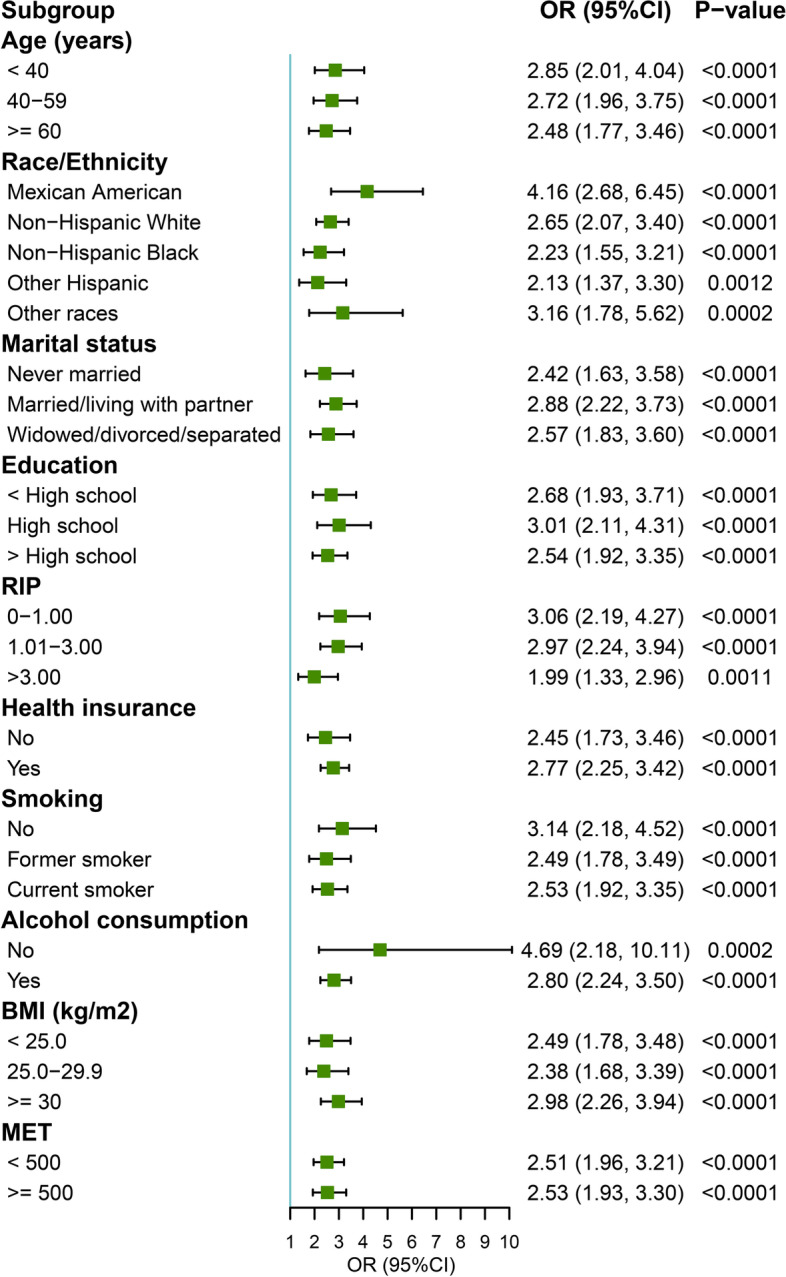
Subgroup analysis of the association between depression and urinary incontinence among male participants ≥ 20 years old in 2005–2018 NHANES. OR, odds ration; CI, confidence interval; RIP, ratio of family income to poverty; BMI, body mass index; MET, metabolic equivalent

As shown in Fig. 3, subgroup analysis was performed to assess the UI-depression correlation stratified by age, race/ethnicity, marital status, education, health insurance, smoking, alcohol consumption, PIR, BMI, and MET. All variables except stratification factor were adjusted in the subgroup analysis. The results showed that compared to men without UI, more than twofold higher odds of depression can be found in men with UI in almost each subgroup. These analyses confirmed that UI was associated with a higher odd of depression in any population.

## Discussion

UI is a very bothersome and embarrassing symptom that is often considered a normal part of aging and causes a significant decrease in quality of life and a huge financial and medical burden [16, 21, 22]. This study, conducted on men over the age of 20 in 2005–2018 NHANES data, systematically analyzed the association between depression and UI type and severity in a large scale of population. The results showed that men suffered from UI had higher odds of depression than those without UI. The odds of depression increase with the severity of UI. Furthermore, among the three UI types, MUI had the highest odds of depression and UUI had the lowest odds. Subgroup analyses showed that men suffered from UI had higher odds of depression in each subgroup.

The association between UI and depression has been investigated in previous studies [16, 23]. An observational cohort study reported that major depression was more common in UI patients than in non-UI patients [24]. Furthermore, moderate and severe UI were related to higher odds of major depression [24, 25]. In this study, higher odds of depression were observed among participants with moderate and severe UI, which was consistent with previous studies. Furthermore, as shown in Fig. 2, the severity of depression is consistent with the severity of UI. This phenomenon strongly indicated the linear relationship between depression and urinary incontinence. As previous studies reported, UUI was the most common type of UI in men [26, 27]. Some studies reported that MUI was associated with the highest prevalence of depression among all UI types in women [28], but few studies have reported this in men. In this study, we found that men with MUI had the highest odds of depression compared to other types of men. And UUI had the lowest odds of depression. This phenomenon is consistent with women. Some factors may be the explanations of this phenomenon. UI can significantly impact the quality of life and is embarrassing for those who need to be in public. Prolonged UI leads to an increased incidence of depression. For different types of UI, MUI is related to the highest odds of depression, but UUI is correlated with the lowest odds of depression. This phenomenon may be related to the degree to which UI interferes with daily activities. For men suffered from UUI, UI can be reduced by increasing the frequency or speed of toilet visits. For MUI, more daily activities are restricted, such as running, jumping, and even laughing, which makes people feel a loss of freedom.

Many factors play roles in the prevalence of depression. Different race/ethnicity may have different prevalence of depression [29]. In this study we reported that compared to non-Hispanic Black and White, Mexican American showed higher risk for depression. The prevalence of depression decreases with age and education, which may be associated with social experience. Besides, men with higher BMI, no smoking or alcohol consumption, and lower family incomes were more likely to develop depression. In general, patients with UI had higher level of depression than those without UI, regardless of any subgroup.

Some explanations for the association between UI and depression are the same physiological factors, such as serotonin (5-HT) or noradrenergic system [30–32]. Although UI can significantly reduce work productivity and quality of life, many patients do not seek treatments [33–35]. For UI patients, they need to actively seek treatment and pay more attention to their own mental health; for clinicians, it is necessary to pay attention to the screening of depression in UI patients, because these diseases often coexist and may aggravate each other's severity.

UI has high odds of depression. It’s important for clinicians to pay more attention to the suffering of depression in the management of UI patients. Previous studies often focus on the association between UI and depression in women or lack covariates adjustment. A previous study constructed multivariable logistic regression models to calculate the prevalence odds ratio, and observed that moderate/severe UI was significantly associated with major depression (OR 2.7; 95% CI 1.6, 4.0) in men [36]. It was an earlier study based on NHANES data that highlighted the association between UI and depression in men. In this study, to further explore this association, the ORs were confirmed by constructing multivariable logistic regression models with partial and all covariates adjustment. The ORs of different UI types and severity were calculated separately. Subgroup analysis and linear relationship analysis were also carried out. Then, we found a linear relationship between depression and urinary incontinence, which has not been reported by previous studies. There are some limitations in this study. Since this was a cross-sectional design, the causality between UI and depression could not be determined. Longitudinal analysis may help reveal whether UI contributes to depression, or the opposite, or the two symptoms coexist by a coincidence. Due to the lack of information on prostate disease (such as the treatment for prostate cancer) in some periods NHANES data, it was not adjusted in this study. Other variables that may influence the association between UI and depression may not be included in this study. Moreover, more cohorts were needed to validate the results.

## Conclusion

Among men, higher odds of depression were observed in patients with UI than those without UI. Besides, the odds of depression increase with the severity of UI. Among the three UI types, MUI had the highest odds of depression and UUI had the lowest odds. It’s necessary to screen depression in UI patients.

## Data Availability

The datasets generated and/or analysed during the current study are available in the NHANES (https://www.cdc.gov/nchs/nhanes/).

## Abbreviations

UI: Urinary incontinence
NHANES: National Health and Nutrition Examination Survey
OR: Odds ratio
CI: Confidence interval
SUI: Stress UI
UUI: Urge UI
MUI: Mixed UI
RIP: Ratio of family income to poverty
BMI: Body mass index
MET: Metabolic equivalent
